# Does Fish Farming Improve Household Nutritional Status? Evidence from Bangladesh

**DOI:** 10.3390/ijerph19020967

**Published:** 2022-01-15

**Authors:** Romaza Khanum, Petra Schneider, Muhammad Salim Al Mahadi, Mohammad Mojibul Hoque Mozumder, Md. Mostafa Shamsuzzaman

**Affiliations:** 1 Department of Agricultural Economics and Policy, Sylhet Agricultural University, Sylhet 3100, Bangladesh; romaza.aep@sau.ac.bd; 2 Department for Water, Environment, Civil Engineering and Safety, University of Applied Sciences Magdeburg-Stendal, Breitscheidstraße 2, D-39114 Magdeburg, Germany; petra.schneider@h2.de; 3 Planning, Development and Works, Sylhet Agricultural University, Sylhet 3100, Bangladesh; mahadi.salim@yahoo.com; 4 Fisheries and Environmental Management Group, Helsinki Institute of Sustainability Science (HELSUS), Faculty of Biological and Environmental Sciences, University of Helsinki, 00014 Helsinki, Finland; mohammad.mozumder@helsinki.fi; 5 Department of Coastal and Marine Fisheries, Sylhet Agricultural University, Sylhet 3100, Bangladesh

**Keywords:** nutritional status, food consumption, calorie intake, fish culture, poverty indices

## Abstract

In the present study, nutritional status was assessed using dietary diversity of fish and non-fish farming households in Mymensingh district of Bangladesh. It has determined the incidence of poverty in fish and non-fish farm households through a comparative analysis of family profile, food consumption, calories, and protein intake. A total of 420 farms were selected for data collection using structured questionnaires with 210 fish and 210 non-fish farm families. The study using both descriptive and functional analysis revealed that the respondent age of both farms was 45.10 years, family size was 5.70, average education was 4.64 schooling years, and average farm size was 0.514 hectares. As a result, due to the increase in household income, fish farm families improved their food consumption, calories, and protein intake in comparison with non-fish farms. On a direct calorie intake (DCI) basis, the overall absolute and hardcore poverty levels of fish farm households were 32 percent and 18 percent, respectively, while those of non-fish farm households were 22 percent and 10 percent, respectively. Therefore, the incidence of poverty was higher in non-fish farming families than in fish farming families. In principle, provision of various forms of government assistance through the Department of Fisheries (DOF) will further intensify and strengthen fish farming, which will easily bring fallow and uncultivated lands of the area under fish farming. Moreover, it is possible to inspire the younger generation through this research that will help them to become a fish farm-based entrepreneur. The main conclusion of the present study is that fish farming is more positively related to household income, family food intake, and nutritional status than any other type of farming.

## 1. Introduction

Food consumption generally refers to the dietary habits of people, which vary from country to country or region to region. Dietary habit is generally assessed through food items and their shares in total consumption, frequency of intake, and nutrient composition. Adequate amount of food consumption both in terms of quantity and quality is one of the key determinants of nutritional status for a human to survive, which results from the relationship between nutrient intake and requirements and from the body’s ability to digest, absorb, and use these nutrients [1]. Adverse outcomes such as low birth weight, malnutrition disability, poor quality of life, and mortality are also related to poor eating pattern [2], which is measured in terms of calorie and protein/nutrient consumption considering input for health production functions [3], while calorie intake is correlated with other nutrients. Accurate measurement needs to be recorded through on-site observations of normal food consumption behavior over a certain period of time [4]. According to the FAO [5], each household member’s nutrient intake is estimated through distributing the household’s food to its members in direct proportion to each member’s share of the household’s total energy requirements, expressed as adult male consumption equivalents (AMEs).

Despite steps taken to reduce global malnutrition by increasing food grain production, people around the world are still suffering from malnutrition, and Bangladesh is no exception. About 36% of children under five years of age are stunted, 33% of children under five years are underweight, 14% of children are wasted, 24% of pregnant women are suffering from anemia, and about 19% of 15- to 19-year-old adolescent girls are malnourished [5] in Bangladesh. Between 2000 and 2015, the total per capita food consumption (intake) in Bangladesh increased slightly (by 6.9%). A significant improvement in food consumption and behavior has been noticed since 1992, and per capita daily calorie intake increased from 2266 kcal in 1991–1992 to 2455.4 kcal in 2015, and the protein consumption increased from 62.72 g in 1991–1992 to 66 g in 2015 [6]. By providing enhanced income, food security, and gender equity, agriculture affects the health and nutrition of poor households in very tangible ways.

As Bangladesh is an agricultural country, its vast water and land is being used for the production of grains, vegetables, fish, livestock, and poultry, which ensures regular food security of the people. However, grain and vegetable production are becoming riskier day by day, due to production uncertainty, low prices, marketing uncertainty, and high price of seeds and fertilizers, etc. Proper use of land may be one of the factors involved in solving grain and vegetable production problems. For the past two decades, land has been used extensively, meaning that it has been used not only for crops but also for non-crop farming, such as fish, poultry, and cattle. In this regards, flood-free cropland area is being converted into freshwater fish farming [7]. In addition, fish farming is more productive than crops [8] and an important food source of animal protein, accounting for about one-fifth of the global animal protein intake [9]. According to a FAO report (2018), Bangladesh achieved self-sufficiency in fish production with per capita fish consumption of 62.58 g/day against a set target of 60 g/day. In order to meet domestic consumption by increasing fish production in the country, the government is also focusing on how to bring ponds and water bodies under fish farming throughout the year. At present, there are 143 government and 1038 private fish farms throughout the country [10]. As a result, crop land has been shifted and is used for fish culture such as pond fish farming, shrimp farming (brackish water aquaculture), and golden prawn farming [11]. Thus, can such a change ensure food security by providing the necessary food to the people of the country?

In this context, the present study also focuses on crop farming, and thus the question is, which agricultural activity plays an important role in ensuring food security as well as family nutrition in the rural economy? Thus, it was very important to find out whether the food consumption and nutritional status of the fish farming family has improved compared to other farms in a particular area. Specifically, the study focused on a comparative review of family conditions in fish and non-fish farms, considering household income and expenditure; both types of farms determined the nature of food intake, calories, and protein intake on the basis of family dietary diversity, and above all, the incidence of both types of poverty in both farm families was estimated on the basis of family income and calorie intake.

## 2. Review of the Literature

In the nutrition literature, one of the common indicators of the quality household diets is ‘dietary diversity’. Dietary diversity is defined as the variety of foods across and within food groups consumed over a given reference period to ensure the required intake of essential nutrients for being in a state of good health [12,13,14]. Thus, a systematic review on the contribution of crops and fish farming to family nutrition shows that only a limited number of studies have been conducted on this method. Few empirical studies [15,16,17] have found a positive relationship between crop production and dietary diversity and three pillars of food security (availability, access and utilization), household nutrition and food security [18,19,20,21,22,23], and ‘direct and important linkage with dietary diversity and nutrition of household members’ [24,25,26], wherein Anderman et al. [27] obtained a negative relationship between cash crop production (cacao and palm oil) and household food security. Aweke et al. [28] found that the size of landholding and farm income are strongly associated with food consumption, diets, and nutrition status, which partly relies on other components of the food systems.

On the other hand, small-scale aquaculture has been recognized as an important opportunity for improving a household’s calorie intake, dietary diversity, and quality of diets in developing countries [29,30]. Many studies [31,32,33,34] have shown that pond-based aquaculture increases household consumption and total energy intake, higher energy intake, and lower levels of undernourishment with fish ponds [32]; the role of homestead aquaculture for household nutrition in Bangladesh [35,36,37]; homestead ponds as a cheap, regular, and easily accessible source of animal protein and essential micronutrients [38]; and relationship to increased purchasing power from selling fish, which helps to purchase more food from the market and improves the quality of household’s diet [39]. Some scholars [40,41] estimated the effects of aquaculture income on household food consumption, dietary diversity, and the quality of household diets with protein rich and energy-dense food items. Production technology and knowledge [42], as well as employment, income, and standard of living [43], are assessed through fish farming, and fish farming is a source of providing micronutrients, nutritious food, and female empowerment [44].

The present study contributes to the literature in several ways. Most of the previous studies have discussed the relationship between agricultural production diversity, crop diversity, household food diversity, and food security. The main focus of their research was only on crops and vegetables, whereas the non-crops, i.e., fish and animal products that are gradually contributing to both nutrition and market income, were not clearly highlighted. This study provides an opportunity for subsequent researchers to broaden the most effective process in fish and crop farming. Moreover, some scholars [45,46] used panel data for analyzing a causal relationship between them, while others [12,47,48,49] have used cross-sectional data and simple econometric methods to analyze potential correlations. However, the present study used strong econometric methods to analyze functional relationships with a better indication of the overall dietary diversity of the food range over a seven-day period, which was absent in other studies. Besides these, this study explains how fish farming activities are more capable of alleviating household poverty than non-fish farming, which is different from other studies. The first shortcoming in this study was to measure the household nutrition on the basis of calorie and protein intake from only one region of Bangladesh. The second drawback was to ask farmers to remember what they ate last week. In the case of research, it was difficult to explain the real picture with this kind of recall data.

## 3. Conceptual Framework: A Household Model

Nutrition is directly related to food intake and household income. Here, the empirical analysis of the econometric model follows the specification of the household model, which reflects the economic situation and the food intake of the households. The following diagram is a conceptual framework of the household model specification for nutritional status taken from Chung [50].

**Figure 1:**
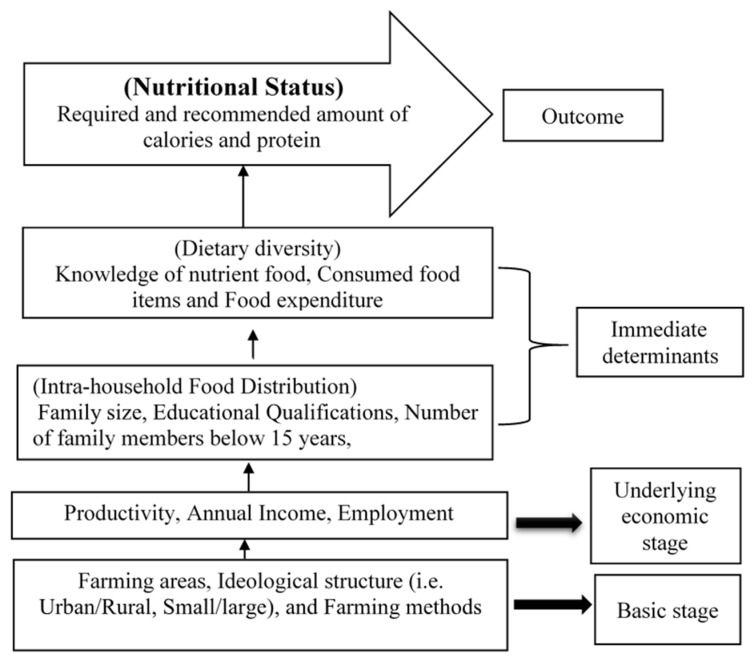
Conceptual framework for nutritional status (sources: Adopted from Chung 2012).

Here, farming income was specified for the household model, which was divided into different levels such as basic, economic, immediate, and outcome. The issues that were considered at the basic level were farming areas such as urban or rural, the type of ideological structure such as small or large, and farming methods, etc. Issues such as productivity, annual income, and employment were considered at the economic level. Immediate determinant stage was divided into intra-household food distribution and dietary intake. First of all, family size; educational qualifications; number of children under 15 in the family, etc.; and knowledge of nutrient food, consumed food items, and food expenditure were considered to obtain the number of calories and protein intake at the household level (Figure 1). In the final stage, taking nutritious food ensured the required and recommended number of calories and protein for each member of the family.

From the conceptual framework, it can be explained that the productivity of farms helps to directly increase the annual income and indirectly secure the household food and nutritional status. Therefore, this conceptual framework of studying nutritional status was a dynamic model that might be altered and expanded through further research. Therefore, it is necessary to know how and to what extent the farming activities provide the recommended calories per capita per day of the farm households. Farming activities are a source of income that create employment. The received income helps to meet the basic needs of the family members, including necessary nutritious food, but the food content and the intake process depends on the regional structure such as rural/urban.

## 4. Methods and Materials

### 4.1. Study Areas and Sampling Techniques

**Figure 2:**
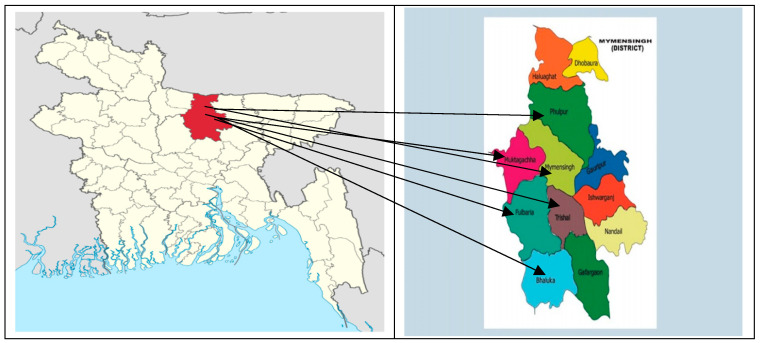
Map of Mymensingh district and selected upazilas in Bangladesh (https://en.wikipedia.org/wiki/Mymensingh_District (accessed on 24 November 2021)).

Mymensingh is known as the greater district of fish farming in Bangladesh. Most areas of the district are flood free, and therefore it is very easy to produce fish in ponds. In 2017–2018, the total fish producing area was 28,620 hectares with 12.50 MT/ha [51]. Due to the growing demand for fish, homestead ponds in that area have come under more intensive cultivation, which helps in increasing the cash income of poor farmers along with food supply. According to the intensive fish farming system, Mymensingh district is divided into lower, medium, and higher intensive fishing zones. Subsequently, a total of six upazilas were selected, with two upazilas from each intensive fishing zone, namely, Bhaluka, Trishal, Muktagachha, Fulbaria, Phulpur, and Mymensingh Sadar (Figure 2). The old proverb *‘Machee Bhatee Bangali’* means ‘fish and rice make bangali’, which intrinsically indicates the importance of both fish and rice. On this basis, two major aspects of the agricultural sector such as fish and crop farming were given priority in village selection. At this stage, a total of 42 villages were selected from each upazila, with 7 villages each. After that, two types of farm households were identified, such as (a) those involved in fish farming and (b) those involved in crop production but not involved in fish culture. To create a comparative perspective between fish and non-fish farm households, we randomly selected 210 farm households from each group, but 5 fish and 5 non-fish farm households were taken from each village. Consequently, the sample size stood at 420.

Both quantitative and qualitative approaches were used for the data collection. Key informant interviews, focus group discussion (FGD), and face-to-face interviews were also used to collect primary information. A total of 20 FGDs were conducted, which were divided into 10 sessions with 21 respondents for each group. The schedule of structured and semi-structural interviews was prepared to reach the objective of research. A structured interview schedule was prepared with open and close questions. Books, journals, annual reports, and internet documents were used as secondary sources. To analyze data, we employed both descriptive and analytic methods.

### 4.2. Measuring Determinants of Farm Households

Dietary diversity and internal family food distribution ensure direct household food security. Here, the heads of the family determine what kind of food is needed to ensure household food security and nutrition. Specifically, the study used three variable indicators, namely, household income, food intake, and dietary diversity, which were calculated in the following ways:

#### 4.2.1. Income of Farm Households (Fh_inc_)

Total household income is usually divided into two parts, namely, agricultural and non-agricultural income. If the lion’s share of the family’s income comes from farms (i.e., fish, crops, and other sources), then the income of that family is usually dependent on agriculture. To calculate farm income, we used the total market value of each source on the basis of farmer estimates, including home consumption and market sales.

$$\mathrm{Percentage}\;\mathrm{of}\;\mathrm{farm}\;\mathrm{income}\;({\mathrm{Fh}}_{\mathrm{inc}})=\frac{{\mathrm{Incf}}_{h}}{{\mathrm{TotInc}}_{h}}$$

where Incf is the household’s income of farm households, TotInc is the total household income, and h represents the household.

#### 4.2.2. Indicators of Food Consumption and Calorie Intake (*Y*_1_ and *Y*_2_)

The household’s food consumption was calculated both in terms of the value of food consumed and the corresponding intake of calorie from those food items. Per capita food consumption (*Y*_1_) is the total value of food consumed in a household divided by its household size, which is calculated by using equivalent scale of households [52]. It is measured in annual terms and in monetary value, which is expressed as

$$Y_{1,\;pc,h}=\frac{1}{HS_{h}}\;\overset{n}{\underset{i=1}{\sum }}EXP_{h,i}$$

where *EXP* is the value of the household’s annual food consumption, *HS* is the household size, *i* represents the farms, and *h* represents the household.

The calorie intake per capita per day (*Y*_2_) is calculated by converting the quantities of the food items consumed to calorie using standard conversion factors suggested by FAO [53]. The sum of the calorie across all food items was divided the household size and 365 days to determine the daily per capita calorie consumption. It was measured in annual terms and is expressed as follows:

$$Y_{2,pcd,h}=\frac{1}{365\times HS_{h}}\sum_{i=1}^{n}KCal_{h,i}$$

where *Kcal* is the calorie consumed from different food items, *HS* is the household size, *i* represents the farm, and *h* represents the household.

#### 4.2.3. Food Consumption Score (*Y*_3_)

The dietary diversity was calculated from the number of different foods or food groups consumed by a household within a specific reference period [54]. A household’s economic ability to consume a set of nutritionally diverse food items was measured by dietary diversity. However, it is only a qualitative figure and thus, to capture nutrition intake accuracy, the World Food Programme (WFP) suggests using the food consumption score (FCS). The FCS captures both a household’s dietary diversity and the consumption frequency of different foods [55]. It assigns a weight to each food item to determine the richness of the consumed food groups, which is important for determining the quality of the household dietary diversity. It captures the richness of consumed food items in the households.

The food consumption score (*Y*_3_) is a composite score based on the household’s dietary diversity, the frequency of food consumption, and the relative nutritional importance of the different food groups. To calculate FCS, we categorized food items consumed by a household into 15 different groups (Table 1). The consumption frequency of each food group was then multiplied by the assigned nutrient-based weights proposed by the WFP [55]. All the values of each food group were then summed to generate the FCS, which is expressed as

$$Y_{3h}=\overset{15}{\underset{i=1}{\sum }}f_{h,i}\times w_{i}$$

where *f*(*h*,*i*) is households’ frequency of consumption of food group *i*, *w_i_* is the weight attributed to each food group, *i* represents the food group, and *h* represents the household.

### 4.3. Modeling and Inferential Statistics

Some statistical models relating to food consumption were estimated, encompassing essential explanatory variables. Descriptive statistics were applied to determine the average, sum, ratio, percentage of data, and the normality test to determine the distribution of calorie intake in both households. Regression analyses with linear and log linear models were used. Specifically, income, expenditure, consumption, and nutrition functions were estimated in both linear and log linear forms. To measure poverty indices, we used the Foster–Greer–Thorbecke (FGT) method [56]. Accordingly, poverty indices were calculated using direct calorie intake (DCI), poverty line estimation, or cost of basic needs (CBN) methods. Daily per capita calorie intake was calculated on all food items using food conversion ratios. Individuals whose daily per capita calorie intakes are less than 2122 kcal and 1805 kcal are said to be under the absolute and hardcore poverty lines, respectively. Furthermore, F-test was carried out by using SPSS software.

Linear income function

$$Y_{i}=\beta_{0}+\beta_{1}X_{1}+\beta_{2}X_{2}+\beta_{3}X_{3}+\beta_{4}X_{4}+\beta_{5}X_{5}+\beta_{6}X_{6}+U_{i}$$

Log-linear income function

$$lnY_{i}=\beta_{0}+\beta_{1}lnX_{1}+\beta_{2}lnX_{2}+\beta_{3}lnX_{3}+\beta_{4}lnX_{4}+\beta_{5}lnX_{5}+\beta_{6}X_{6}+U_{i}$$

where *Y* = total household’s income (Bangladeshi currency, BDT) from all sectors in a year, $X_{1}$ = income from fish/crop, $X_{2}$ = income from other sources, $X_{3}$ = farm size (hectare), $X_{4}$ = family size, $X_{5}$ = age of the household head (year), and $X_{6}$ = education (year of schooling). Note: education is used without log in the log-linear model. Similar functions were formulated and estimated for household expenditure, food consumption, calories, and protein intake.

### 4.4. Poverty Estimation

The Bangladesh Bureau of Statistics (BBS) used the following semi-log or exponential model to estimate the poverty line:

$$\ln Y=\beta_{0}+\beta_{1}X+U$$

where *Y* = per capita monthly expenditure (food and non-food), *X* = per capita per day calorie intake, and *U* = disturbance term.

## 5. Result and Discussion

### 5.1. Results of Household Profile

The purpose of households’ profile was to identify the age of the head of the family, educational qualifications, farm size, number of family members, number of earning adults, and number of children under 15 years of age.

Table 1 shows that the overall average age of farm household head was 45.10 years, which was insignificant variations (F = 0.033). Heads of the fish farm households were comparatively young and educated with 7.71 years of schooling, being significant at the 1% level. According to the family data, all the farm households had their own land at an average of 0.514 hectares, with a significant difference of 5.70 persons (F = 5.108). The average number of adult members of the household (4.10) was at a significant level (F = 3.34), and the number of members under 15 was only 1.60. The results show that the number of adult members in a fish farm household was slightly higher than that of a non-fish farm household.

### 5.2. Household Income and Expenditure

A total of eight types of income sources were identified for determining the annual income in both fish and non-fish farm households. These were crops, vegetables, fish farming, poultry, livestock, business, services, and other sources. Here, other sources of income included house rent, rental cars, machinery and agricultural equipment, and workers’ wages. Fish and crops contributed the most to the annual income of the total household income at a significant level.

Table 2 shows that annual income of both the farm households was significant at the level of 1%. However, fish farm households were well-equipped and significant in both linear and log-linear for fish farming and other sources of income, whereas non-fish farm households were quite good in terms of crop production and significant in terms of farm size as well as other sources of income. Table 2 further explains that the non-fish farm household size was significant at the 1% levels in linear and log-linear form, but in both forms, it had a negative relationship with the fish farm households.

Table 3 presents a total of 10 items of annual household expenditure, namely, food, medical expenses, clothing, housing, education, festivals, transportation, mobiles, electricity, and others. The study found that farm households spend a large portion of their household expenses on food, followed by education, festivals, clothing, and medical expenses. Overall, farm households spend about 30 percent of their total expenditure on food, whereas fish farm households spend about 28 percent and non-fish farm households about 41 percent.

On the other hand, Table 3 shows that the total food expenditure of both types of farm households accounted for 36% of total income, whereas fish farm households spent 33% of total income, and non-fish farm households, 42 percent. In comparison, the number of fish farm household members was high, which increased the cost of food, but the educational qualifications affect the integrated food system, which is difficult for non-fish farm households. Moreover, fish production in the pond ensures the intake of animal protein. Thus, it can be said that food is one of the most important components of household expenditure, although food expenditure of fish farm family is less than that of non-fish farm family. This result is consistent with the research of Rahman and Sausa-Poja (2010) [57], where it was said that farmers spend the lion’s share of their total income, i.e., about 43%, on food.

Table 4 analyzes the factors influencing food expenditure in both types of households. In the case of overall annual household expenditure, food expenditure was positive and significant at the 1% level. It was statistically proven that household size has significant but negative effects on both household spending. This means that household expenditure is not determined by the number of family members. Table 4 further proves that education spending was positive and significant at the 1% level, as farm families were more conscious and interested than ever in spending on their children’s education.

### 5.3. Intake of Food by Food Items

The per capita food intake of fish and non-fish farm households for 2019–2020 is presented in Table 5. The individuals consumed a variety of food items that are similar and consistent with HIES [58], with a few exceptions. Fifteen food items were identified, wherein rice was the most important, followed by vegetables and tubers. Here, the food intake of the families is explained in detail, considering the main food items.

The per capita amounts of rice for both fish and non-fish households were 419 and 412 g, which was comparatively higher than the national average (367.19 g) [58]. The second important food option was vegetables, where fish and non-fish farm families consumed about 100.06 and 969 g per capita per day, but this was comparatively lower than the national average (167.3 g) [58]. Overall, Table 5 shows that per capita per day food intake was higher in fish farm households than in non-fish farm households. Therefore, the total per capita per day food consumption of both farm households was 966.54 g, whereas fish farm households received 1068.08 g and non-fish farm households received 864.98 g. While 25 percent of the fish farm households consumed 1055 g of food per day, 75% of the non-fish farm households consumed only 871 g of food, which was much less than the fish farm households.

### 5.4. Calorie Intake by Food Items

Calorie consumption per capita per day divides the daily caloric supply in the household according to the size of the household [59]. Household caloric availability was estimated from food nutrition, which was measured in kilocalorie units. Each food item has its own caloric value, which is completely different from each other. Table 6 shows that the average daily calorie intake of overall farm households was 2066.54 kcal, whereas fish farm households consumed 2245.73 kcal and non-fish farm households consumed only 1892.48 kcal. Considering individual foods, rice was found to have the highest dietary energy (1350.38 kcal), followed by oil and fat (207.10 kcal), wheat (77.76 kcal), meat (68.68 kcal), fish (63.60 kcal), sugar (61.55 kcal), and tubers (55.20 kcal). In the case of fish farm households, oil provided the most calories after rice, but meat and fish provided the same number of calories, which were about 98.91 and 92.22 kcal, respectively. The studies also found that the overall vegetables (21.38 kcal), eggs (21.64 kcal), and fruits (10.34 kcal) were much lower than expected, wherein Raman and Islam (2012) [60] showed comparatively good results. Table 6 shows that the calorie intake rate of the fish farm households (2245.66 kcal) was higher than that of the non-fish farms (1887.43 kcal). The non-fish farm family consumed the most calories from oil after rice, but among other food items, the main one was the tuber, which was about 59.80 kcal.

Normality tests were performed to determine the distribution of calories in both farm households. Both descriptive statistics and graphical presentation showed that calories were normally distributed among family members. However, whereas 75% of non-fish farm households consumed a maximum of 1879.42 kcal per capita per day, the minimum calorie intake of a fish farm household was 2211.03 kcal, which was much higher than the recommended calorie intake. In this case, 75% of the fish farm households consumed a maximum of 2275.8 kcal per capita per day.

### 5.5. Intake of Protein by Farm Households

Protein is an important nutrient element to maintain good health of people. From Table 7, it is shown that the average per capita per day’s protein intake of both farm households was 70.99 g wherein the fish farm household obtains 84.22 g daily. Among the stated food elements, rice (20.77 gm) provided the maximum amount of protein, followed by oils and fats (12.13 g), meat (10.10 g), and vegetables (8.38 g). The fish farm households consumed the most protein from rice, but oils and fats, meat, fish, and vegetables provided 16.25 g, 14.40 g, 8.70 g, and 8.59 g of protein, respectively. On the other hand, non-fish farm households received the highest protein from rice, but as usual, vegetables (8.16 g), oils and fats (8.00 g), and meat (5.80 g) were the main protein supplements. Thus, the table indicated that due to increased income from fish farming, fish farm households were more able to meet family protein needs than non-fish farm families.

### 5.6. Poverty Indices Estimation

The estimated model was

$$\begin{array}{c} lnY_{i}=13.84+\left(-0.002\right)X_{i}\;\\ \;(2.90)\;(0.001) \end{array}$$

$$Adjusted\;R^{2}=0.005,\;F=2.05$$

$$\begin{array}{cc} \mathrm{Absolute}\;\mathrm{poverty}\;\mathrm{line}\;(lnY_{i}) & =13.84+\left(-0.002\right)X_{i}\\ & =13.84-0.002\times 2122\\ & =9.596 \end{array}$$

*Y* = Exp (9.596)

     = Tk. 14705.84

$$\begin{array}{cc} \mathrm{Hardcore}\;\mathrm{poverty}\;\mathrm{line}\;(lnY_{i}) & =13.84+\left(-0.002\right)X_{i}\\ & =13.84-0.002\times 1805\\ & =10.23 \end{array}$$

*Y* = Exp (10.23)

     = Tk. 27722.51

Various food items are provided to ensure the nutrition of the family members by earning cash from the source of household income. Thus, at this stage, it is necessary to determine the poverty line in terms of nutrition and income of fish and non-fish farm households, wherein poverty is one of the foundations of family nutrition. The poverty level was measured on the basis of direct calorie intake (DCI) method using head count ratio and the cost of basic needs (CBN) method using absolute exponential model for Tk. 14,705.84 and Tk. 27,722.51 for fish and non-fish farm households, respectively, as shown in Table 8. The average absolute and hardcore poverty indexes on the basis of DCI were 54 and 35 percent, respectively, which was comparatively lower than that of Ria et al. (2019) [61]. Rising prices of essential commodities generally increase the prevalence of poverty in developing countries such as Bangladesh, which is reflected in this study. The overall absolute and hardcore poverty indexes based on CBN method were, respectively, 53 and 37 percent. Therefore, both methods proved that the incidence of poverty in non-fish farm households is significantly higher than in fish farm households.

## 6. Discussion

The results indicated that the heads of the fish farm household were relatively young and educated, and that the number of family members was much more active than in the non-fish farm families. Some studies have also demonstrated the positive influence of education of household heads on dietary outcomes or nutrition [50,62,63]. In addition, due to the large size of fish farms, their family income is much higher than that of crop farms. So why are family members losing interest in getting involved in crop production? The main reasons are uncertainty in crop production and lack of dignity of the new generation. The new generation considers fish farming as a secured and profitable source, one that ensures family income by increasing direct interest. Fish farming income is much higher than other farms in the research areas, where about 43% of the total income is spent on food. The increase in fish farm income provided 15 types of food items for their family members, which ensured food, calorie, and protein intake. Per capita food consumption among total expenditures was a strong influence in determining the level of household dietary diversity, which agreed with the previous literature [64,65]. The results clearly indicate that the nutritional status of the fish farm households was good because the calorie and protein levels in the diet were higher than the recommended levels. Therefore, fish farming in the research area was a major source of cash income that increased the purchasing power of the farm households. As a result, increased purchasing power directly contributed to ensuring food security and nutritional status by ensuring food diversity.

## 7. Conclusions

As Bangladesh is an agricultural country, it was very important to measure the relationship between farm and household nutritional status by ensuring food security in a particular area of the country. In the study, 420 farms from 7 villages were selected, focusing on 210 fish and 210 non-fish farm households that have been practicing for several years. Thus, we examined whether fish farm households improved the nutritional conditions and dietary diversity better than non-fish farm households. Appropriate statistical tools or techniques such as descriptive statistics, econometric models, normality test, and F-tests were used for partial and functional analysis. In addition, direct calorie intake (DCI) and cost of basic needs (CBN) methods were used to measure nutritional status and poverty incidence. Therefore, after analyzing the results, we found it clear that this study on fish farming was very important for the implementation of nutrition, health, and agricultural policies of rural households in Bangladesh, such as (1) its income ensured food, calories, and proteins through ensuring dietary diversity in the family, which reflects similar results in previous research by Gomna and Rana [65] and Dey et al. [8]; (2) its income is a complementary and significant contribution to other sources of income; (3) income earned from selling fish indicated the advanced socio-economic status of the farm households; (4) its enhanced income can help to create commercial farms from the subsistence; (5) the young generation will be motivated to establish themselves as a successful entrepreneur by taking up fish farming activities; and, finally, (6) its sustainable income encourages rural young farmers to earn a sustainable livelihood, as well as assisting the government in formulating sound policies by ensuring proper use of land. Therefore, it indirectly contributes to the economic growth of Bangladesh through the development of rural infrastructure as a guideline for further research in other parts of Bangladesh.

Studies have shown that fish farming has increased household income and improved per capita per day food consumption, calories, and protein intake compared to non-fish farm households. However, food diversification is needed in rural farming households to improve nutritional status as well as ensure food security. Thus, crop farming is an important agricultural activity without which family nutrition is completely uncertain. Therefore, in order to recommend the policy, one must give equal importance to fish farming as well as crop cultivation. For this, it is necessary to bring the fallow and uncultivated lands under cultivation to diversify the production and not just produce fish or crops. It is very important to determine the land according to the suitability of crop/fish production. Therefore, the recommendation is that the Department of Fisheries needs to reconsider its approach to the role of fish farming in a more recognized way through their extension activities, i.e., to make fish farming more accessible. Moreover, if fish farming is gradually becoming a semi-commercial or fully commercial small-scale fish production system, rapid marketing and improved infrastructure are required.

## Figures and Tables

**Table 1:** Fish and non-fish farm households’ profile.

Farm Households	Age of Household Head (Year)	Educational Level of Household Head (Year of Schooling)	Farm Size (Hectare)	Family Size		
				Total Family Size	Adult Family Members	No. of Family Members below 15 Years
Fish farm	43.33 (9.112)	7.71 (3.595)	0.541 (41.977)	5.81 (1.988)	4.12 (1.929)	1.69 (0.925)
Non-fish farm	46.88 (8.643)	1.57 (1.899)	0.487 (65.14)	5.60 (1.833)	4.09 (1.533)	1.51 (1.004)
All farms	45.10 (9.047)	4.64 (4.207)	0.514 (55.143)	5.70 (1.913)	4.10 (1.741)	1.60 (0.968)
F-values	0.033	80.907 ***	0.147	5.108 **	3.343 *	1.361

Figures in the parentheses indicate standard deviations. *, **, and *** indicate significance at 0.1, 0.05, and 0.01 probability levels, respectively.

**Table 2:** Annual household income function of farm households.

Variables	Fish Farm Households		Non-Fish Farm Household		All Farm Households	
	Linear Model	Log-Linear Model	Linear Model	Log-Linear Model	Linear Model	Log-Linear Model
Intercept	−57,640.11 (38,104.12)	0.424 (0.303)	40,345.01 (16,418.62)	4.87 *** (0.36)	25,010.68 *** (9249.98)	4.21 *** (0.509)
Income from fish/crops	1.00 *** (0.004)	0.907 *** (0.01)	0.93 *** (0.022)	0.26 *** (0.01)	0.998 *** (0.002)	0.542 *** (0.017)
Income from others	0.95 *** (0.06)	0.105 *** (0.008)	0.93 *** (0.018)	0.43 *** (0.02)	0.968 *** (0.010)	0.209 *** (0.019)
Farm size	128.66 (128.87)	−0.069 * (0.039)	3489.16 ** (1662.54)	0.19 *** (0.049)	−44.11 (27.85)	−0.229 *** (0.054)
Family size	−826.60 (2604.87)	0.005 (0.027)	−32.80 (44.00)	−0.086 *** (0.033)	610.06 (1809.70)	0.138 ** (0.062)
Age	1095.29 * (613.67)	0.036 (0.044)	−456.06 (329.38)	−0.081 (0.08)	−137.67 (177.17)	0.175 * (0.10)
Education	435.47 (1561.85)	0.006 ** (0.003)	1640.59 (1486.24)	0.018 ** (0.008)	−915.16 ** (418.99)	0.017 *** (0.006)
Adjusted R^2^	0.996	0.979	0.967	0.848	0.999	0.778
F-value	9882.41 ***	1613.34 ***	1007.64 ***	181.73 ***	76,342.38 ***	236.53 ***

Figures in the parentheses indicate standard deviation; *, ** and *** indicate significance at the 0.1, 0.05, and 0.01 probability levels, respectively.

**Table 3:** Annual households’ expenditure of different items (in Bangladeshi currency, BDT) in 2020.

Cost Items	Fish Farm Households	Non-Fish Farm Household	All Farm Households	F-Values
Food	399,248.6 (372,779.6)	156,390.5 (74,500.12)	277,819.5 (294,729)	145.58 ***
Medical expenses	88,609.52 (97,543.16)	21,347.62 (20,720.68)	54,978.57 (78,063.31)	124.21 ***
Clothing	110,109.5 (136,137.6)	27,090.48 (18,844.78)	68,600 (105,588.3)	194.42 ***
Housing	87,418.09 (299,429.81)	10,607.14 (19,260.72)	49,012.62 (215,373.3)	100.91 ***
Education	159,333.33 (167,085.95)	43,873.81 (33,466.7)	101,603.6 (133,509.9)	153.91 ***
Festival	154,880.95 (231,979.77)	35,345.71 (21,371.45)	95,113.33 (175,076)	0.003
Transport	101,289.52 (111,407.34)	23,250.48 (16,498.06)	62,270 (88,616.66)	141.19 ***
Mobile	38,037.14 (61,016.25)	8634.29 (4601.81)	22,435.71 (45,952.12)	100.09 ***
Electricity	52,941.90 (61,762.93)	7821.43 (9528.52)	30,381.67 (49,580.69)	116.16 ***
Other	246,679.05 (241,606.62)	51,671.43 (41,734.66)	149,175.2 (198,785.6)	108.05 ***
Total	302,147,000	80,688,900	382,835,900	
Share of food cost to total cost	0.28	0.41	0.30	
Share of food cost to total income	0.33	0.42	0.36	

Figures in the parentheses indicate standard deviation; *** indicates significance at the 0.01 probability level.

**Table 4:** Annual expenditure function of farm households.

Variables	Fish Farm Households		Non-Fish Farm Household		All Farm Households	
	Linear Model	Log-Linear Model	Linear Model	Log-Linear Model	Linear Model	Log-Linear Model
Intercept	547,395.69 (374,866.45)	1.447 ** (0.664)	33,490.67 (31,873.73)	−0.321 (0.357)	114,937.16 (165,109.04)	−0.47 (0.326)
Expenditure on food	3.469 *** (0.145)	1.091 (0.031)	2.856 *** (0.087)	1.105 *** (0.029)	3.502 *** (0.10)	1.147 *** (0.021)
Farm size	−3192.77 ** (1257.202)	−0.269 *** (0.085)	−48.756 (87.137)	0.025 (0.026)	−1025.38 ** (492.55)	−0.023 (0.033)
Family size	−66,025.84 ** (26,984.97)	−0.221 *** (0.061)	13,481.26 *** (3510.50)	−0.199 *** (0.043)	−46,399.42 *** (14,498.21)	−0.237 *** (10.04)
Age	4132.479 (6032.44)	0.054 (0.097)	−268.35 (647.0)	0.042 (0.063)	2020.01 (3115.97)	0.028 (0.062)
Education	17,913.798 (15,259.97)	0.008 (0.006)	−1289.04 (2936.5)	−0.003 (0.006)	27,512.29 *** (7137.27)	0.020 *** (0.003)
Adjusted R^2^	0.745	0.875	0.868	0.907	0.791	0.922
F-value	123.015 ***	293.47 ***	275.886 ***	397.289 ***	318.59 ***	968.99 ***

Field survey, 2020; ** and *** indicates significance at the 0.05 and 0.01 probability level.

**Table 5:** Consumption of food items by fish and non-fish farm household.

Food Items Consumed	Fish Farmers		Non-Fish Farmers		All Farmers		F-Values
	Mean	SD	Mean	SD	Mean	SD	
Grain staples Rice Wheat	419 31	8.19 4.63	412 17	1.76 2.47	415.5 24	6.87 7.92	164.20 *** 40.98 ***
Tubers (potato, sweet potato, etc.)	55	3.86	65	4.47	60	6.51	16.35 ***
Green leafy vegetables (*Pui shak/lal shak/palong shak/kolmi/data shak*)	41.01	5.22	29	1.90	35	7.18	127.81 ***
Vegetables (tomato, carrot, gourd, etc.)	101.06	4.40	96	2.98	98.53	4.53	7.66 ***
Pulse (*mug*, *musuri*, *kheshari*, etc.)	16	3.67	10	1.28	13	4.07	250.99 ***
Fruits (mango, banana, jackfruits, and others)	52	4.54	26	2.29	39	13.50	73.99 ***
Meat (beef, poultry)	72	4.44	29	2.01	50.5	21.79	110.86 ***
Fish (fresh fish, dry fish)	87	5.28	33	1.79	60	27.32	110.71 ***
Eggs	10	2.33	18	1.57	14	4.47	21.45 ***
Milk and dairy products	63	2.41	49	2.33	56	7.39	0.97
Oils and fats (soybean oil, mustard oil, ghee)	65	4.95	32	2.26	48.5	16.96	85.24 ***
Sugar/gur/honey	17	2.29	16	1.57	16.5	2.03	42.72 ***
Onion	22	2.49	19	1.28	20.5	2.48	82.65 ***
Garlic	9	1.05	7	1.39	8	1.59	15.06 ***
Chilly	8	1.37	7	1.39	7.5	1.47	0.00
Total food consumed (g/day/capita)	1068.08	18.48	864.98	9.18	966.54	102.71	

Field survey, 2020; *** indicates significance at the 0.01 probability level.

**Table 6:** Calorie intake of food items by fish and non-fish farm households (Kcal/day/capita).

Food Items Consumed	Fish Farmers		Non-Fish Farmers		All Farmers	
	Mean	SD	Mean	SD	Mean	SD
Grain staples Rice Wheat	1361.73 100.44	26.61 14.99	1339 55.09	5.70 8.00	1350.38 77.76	1.92 0.62
Tubers (potato, sweet potato, etc.)	50.60	3.55	59.80	4.11	55.2	0.11
Green leafy vegetables (*Pui shak/lal shak/palong shak/kolmi/data shak*)	12.30	1.57	8.70	0.57	10.5	0.06
Vegetables (tomato, carrot, gourd, etc.)	21.93	0.96	20.83	0.65	21.38	0.13
Pulse (*mug*, *musuri*, *kheshari*, etc.)	52.66	12.08	32.88	4.21	42.78	0.16
Fruits (mango, banana, jackfruits, and others)	14.25	1.24	6.42	0.57	10.34	0.05
Meat (beef, poultry)	97.91	6.03	39.45	2.73	68.68	0.26
Fish (fresh fish, dry fish)	92.22	5.59	34.98	1.89	63.6	0.20
Eggs	17.59	4.10	31.68	2.76	24.64	0.44
Milk and dairy products	41.58	1.59	32.34	1.54	36.96	0.12
Oils and fats (soybean oil, mustard oil, ghee)	277.55	21.12	136.64	9.63	207.10	0.57
Sugar/gur/honey	63.43	8.56	59.66	5.87	61.55	0.11
Onion	10.23	1.16	8.84	0.59	9.53	0.07
Garlic	12.33	1.44	9.58	1.90	10.96	0.11
Chilly	18.97	3.25	16.58	3.29	17.77	0.18
Total calorie intake	2245.73	45.56	1892.48	18.70	2066.54	2.40
Skewness Kurtosis	0.498 0.267		0.198 −0.115			
Kolmogorov–Smirnov test Shapiro–Wilk test	0.044 0.983		0.029 0.994			

Field survey, 2020.

**Table 7:** Protein intake of food items by fish and non-fish farm household (g/day/capita).

Food Items Consumed	Fish Farm		Non-Fish Farm		All Farm Households	
	Mean	SD	Mean	SD	Mean	SD
Grain staples Rice Wheat	20.96 3.75	0.41 0.56	20.60 2.06	0.09 0.29	20.77 2.91	0.34 0.96
Tubers (potato, sweet potato, etc.)	1.10	0.08	1.30	0.09	1.20	0.13
Green leafy vegetables (*Pui shak/lal shak/palong shak/kolmi/data shak*)	3.49	0.44	2.47	0.16	2.98	0.61
Vegetables (tomato, carrot, gourd, etc.)	8.59	0.37	8.16	0.25	8.38	0.39
Pulse (*mug*, *musuri*, *kheshari*, etc.)	3.20	0.73	2.00	0.26	2.60	0.81
Fruits (mango, banana, jackfruits, and others)	0.47	0.04	0.23	0.02	0.35	0.12
Meat (beef, poultry)	14.40	0.89	5.80	0.40	10.10	4.36
Fish (fresh fish, dry fish)	8.70	0.53	3.30	0.18	6.00	2.73
Eggs	1.20	0.28	2.16	0.19	1.68	0.54
Milk and dairy products	1.26	0.05	0.98	0.05	1.12	0.15
Oils and fats (soybean oil, mustard oil, ghee)	16.25	1.24	8.00	0.56	12.13	4.24
Sugar/gur/honey	0.00	0	0.00	0	0	0.00
Onion	0.26	0.03	0.23	0.02	0.25	0.03
Garlic	0.48	0.06	0.37	0.07	0.42	0.08
Chilli	0.13	0.02	0.11	0.02	0.12	0.02
Total protein consumption	84.22	2.13	57.77	0.88	70.99	13.34

Field survey, 2020.

**Table 8:** Poverty indexes of fish and non-fish farm households.

Farm Households	Direct Calorie Intake (DCI) Method		Cost of Basic Needs (CBN) Method	
	Absolute Poverty (%)	Hard Core Poverty (%)	Absolute Poverty (%)	Hard Core Poverty (%)
Fish farm households	32	18	22	10
Non-fish farm households	76	51	83	64
All farm households	54	35	53	37

## Data Availability

Not applicable.
